# Identification of Genes Associated with Seed Weight and Development of Functional Markers for *GmUBP15* in *Glycine max*

**DOI:** 10.3390/biology15090727

**Published:** 2026-05-02

**Authors:** Furui Wang, Huilong Hong, Zhihao Zhang, Jiangyuan Xu, Lili Yu, Suning Li, Yinghui Li, Lijuan Qiu

**Affiliations:** 1 College of Agronomy, Northeast Agricultural University, Harbin 150030, China; 2 State Key Laboratory of Crop Gene Resources and Breeding, Institute of Crop Sciences, Chinese Academy of Agricultural Sciences, Beijing 100081, China

**Keywords:** soybean, 100-seed weight, *UBP15*, haplotype, KASP

## Abstract

Seed weight is a key agronomic trait determining soybean yield and quality, yet only a few of genes regulating this trait have been functionally characterized to date. In this study, we identified 155 homologous genes in the soybean genome through BLAST searches using 78 functionally validated rice grain weight-related genes as queries. Haplotype analysis prioritized 40 candidate genes exhibiting significant differences in seed weight between haplotypes. To further refine the candidate list, we integrated haplotype frequency analysis, expression–trait association mapping, and tissue-specific expression profiling, ultimately delineating eight key genes. Given the established role of ubiquitination in seed development, we focused on homologs of *OsUBP15* and identified three candidate genes, *GmUBP5*, *GmUBP11*, and *GmUBP33*, that exhibited significant haplotype-dependent variation in seed weight. Subcellular localization assays confirmed their nuclear localization. Haplotype frequency analysis revealed that the superior haplotypes of these genes have been preferentially retained during modern breeding and are widely distributed across major soybean-producing regions. Leveraging non-synonymous SNP variants, we developed and validated robust KASP markers that efficiently discriminate germplasm with contrasting seed weight phenotypes. Collectively, our study provides not only high-confidence genetic targets and actionable molecular markers but also insights into pyramiding breeding strategies for improving seed weight in soybean.

## Simple Summary

Seed weight is an important trait for soybean yield and quality, but few of the genes controlling it are well understood. In this study, we searched the soybean genome for genes related to seed weight, using known rice genes as a guide. We narrowed down our list and focused on three genes that showed strong links to seed weight variation. These genes are located in the cell nucleus, and their favorable forms have been selected for during modern breeding. We also developed simple molecular markers that can help breeders quickly identify plants with heavier seeds. Our findings provide useful genetic targets and practical tools to improve soybean yield through breeding, which can benefit farmers and increase food production.

## 1. Introduction

Soybean (*Glycine max* (L.) Merr.) is an important oilseed and forage crop with a domestication history spanning over 5000 years, representing one of the earliest crops cultivated by humans [1]. Seed weight, typically measured as hundred-seed weight, serves as a key determinant of soybean yield. Although nearly 400 quantitative trait loci (QTLs) associated with hundred-seed weight have been cataloged in the SoyBase database (https://legacy.soybase.org/, accessed on 30 August 2025), only a limited number of underlying functional genes have been molecularly characterized to date.

The genetic and molecular mechanisms underlying grain weight in rice have been extensively characterized. To date, nearly 500 grain size-related QTLs have been found on the 12 chromosomes, and approximately 100 genes regulating grain size have been cloned [2]. In contrast, research on genes associated with soybean seed weight has lagged behind. Therefore, drawing on research findings from crops such as rice to identify key regulators of soybean seed weight and elucidate their molecular mechanisms is of critical importance.

Existing research indicates that rice grain weight is regulated by the coordinated action of multiple signaling pathways, primarily including plant hormone signaling pathways, transcriptional regulatory networks, the mitogen-activated protein kinase (MAPK) cascade, and the ubiquitin-proteasome pathway [3,4]. For example, OsARF4, a transcriptional repressor in the auxin signaling pathway, negatively regulates grain size, as its loss of function leads to enlarged rice grains [5]. Similarly, knockout lines of the squamosa promoter-binding protein OsSPL18 exhibit significantly reduced grain width and thickness, resulting in decreased thousand-grain weight [6]. In addition, Mitogen-activated protein kinase kinase OsMKK4 and its downstream target OsMAPK6 play critical roles in regulating rice grain size by modulating cell proliferation, brassinosteroid signaling, and hormonal homeostasis [7,8].

The ubiquitin-proteasome pathway regulates the stability of growth-related proteins by coordinating cell proliferation and cell enlargement, thereby orchestrating the development of maternal tissues, the embryo, and the endosperm. Through a complex network of factor interactions, it ultimately determines seed size [9]. Ubiquitination is a reversible dynamic process, and the hydrolysis of ubiquitin chains is catalyzed by deubiquitinating enzymes (DUBs) [10]. The ubiquitin-specific protease OsUBP15 regulates seed size by influencing cell proliferation; seed size and weight are significantly reduced in its loss-of-function mutants. Similarly, Arabidopsis *AtUBP15* and Brassica napus *BnaUBP15s* positively regulate seed size and weight [11,12,13]. Ubiquitin-specific protease 15 (UBP15) is a deubiquitinating enzyme that plays a conserved and important role in regulating plant seed development. However, the *UBP15* genes in soybean remain functionally uncharacterized to date.

In this study, we identified soybean homologs of rice grain weight regulatory genes. Through integrated haplotype, frequency, and expression analyses, we screened candidate genes and focused on the homologs of *OsUBP15* for further functional characterization. Based on non-synonymous SNP sites, we developed corresponding functional molecular markers. This work provides both a theoretical basis and practical tools for high-yield molecular breeding in soybean.

## 2. Materials and Methods

### 2.1. Plant Materials and Phenotyping

A total of 1100 soybean germplasm accessions were used in this study. These accessions were provided by the State Key Laboratory of Crop Gene Resources and Breeding, Institute of Crop Sciences, Chinese Academy of Agricultural Sciences. They were cultivated in Jiangxi and Anhui provinces, China, during the 2017–2018 growing seasons, and hundred-seed weight was measured following standard protocols.

### 2.2. Statistical Analysis

Phenotypic data from the soybean germplasm accessions were processed using Excel 2019. Best linear unbiased predictions (BLUPs) were calculated using the lme4 package in R (version 4.4.2) [14]. Statistical analyses of numerical data were performed using GraphPad Prism 10.0 and SPSS (version 19, IBM, Armonk, NY, USA). Differences between groups were evaluated by one-way ANOVA, with details of the statistical tests provided in the figure legends. Statistical significance was defined as *p* < 0.05.

### 2.3. Identification of Grain Weight-Related Genes in Rice and Soybean

In this study, 78 functionally validated rice grain weight-related genes were used as queries to perform BLAST searches against the Phytozome 13 databas (https://phytozome-next.jgi.doe.gov/, accessed on 15 July 2025). For each query, the two soybean homologs exhibiting the highest sequence similarity were retained. After removing sequences with missing data or duplicates, a total of 155 soybean candidate genes associated with seed weight were identified. Based on their chromosomal positions, a physical distribution map was generated using the MG2C online platform (http://mg2c.iask.in/mg2c_v2.1/index.html, accessed on 5 August 2025). All gene names are listed in Supplementary Table S1.

### 2.4. Expression Profiling of Candidate Genes in Soybean

Expression levels of the candidate genes in different soybean tissues were retrieved from the Soybase database (https://www.soybase.org/, accessed on 30 August 2025). Tissue-specific expression patterns were visualized and expression profiles were constructed using TBtools-II (v2.452) software [15].

### 2.5. Retrieval of UBP15 Homologous Sequences in Soybean

Using the OsUBP15 amino acid sequence as a query, we performed BLAST searches against the soybean genome in the Phytozome 13 (https://phytozome-next.jgi.doe.gov/, accessed on 30 September 2025). Homologous genes with sequence identity greater than 50% were retained. Gene nomenclature followed the established naming conventions for members of the soybean UBP gene family [16].

### 2.6. Subcellular Localization of GmUBP5, GmUBP11, and GmUBP33

The coding sequences (CDS) of *GmUBP5*, *GmUBP11*, and *GmUBP33* were amplified by PCR using cDNA from soybean cultivar Williams 82 as a template and individually cloned into the pCEP01-linker-GFP vector. The resulting recombinant plasmids and the empty vector were separately transformed into *Agrobacterium tumefaciens*, cultured, and infiltrated into *Nicotiana benthamiana* leaves. All samples were co-infiltrated with a red fluorescent nuclear marker for nuclear signal identification. After 48 h of incubation, both GFP fluorescence and nuclear marker signals were observed using a laser scanning confocal microscope (LSM980, ZEISS, Oberkochen, Germany).

### 2.7. KASP Marker Development and Validation

Specific KASP probes were designed based on six SNP sites: GmUBP5-39948753, GmUBP11-8094856, GmUBP11-8095604, GmUBP11-8095691, GmUBP33-36419360, and GmUBP33-36413592 (Supplementary Table S2). Primer pairs were designed according to LGC Genomics standards, with flanking sequences obtained from the *Glycine max* Wm82.a2.v1 reference genome. Genomic DNA was extracted from 54 soybean accessions. Reaction systems and PCR protocols were prepared following the instructions for the 2× Master Mix reagent (Chengdu Hanchen Guangyi Biotechnology Co., Ltd., Chengdu, China). FAM and HEX were used as reporter fluorophores, with ROX serving as the reference fluorophore. Fluorescence signals were detected using the QuantStudio 7 Pro system, and KASP genotyping data were analyzed accordingly (Supplementary Table S3).

## 3. Results

### 3.1. Identification of Candidate Genes Governing Seed Weight in Soybean

**Figure 1:**
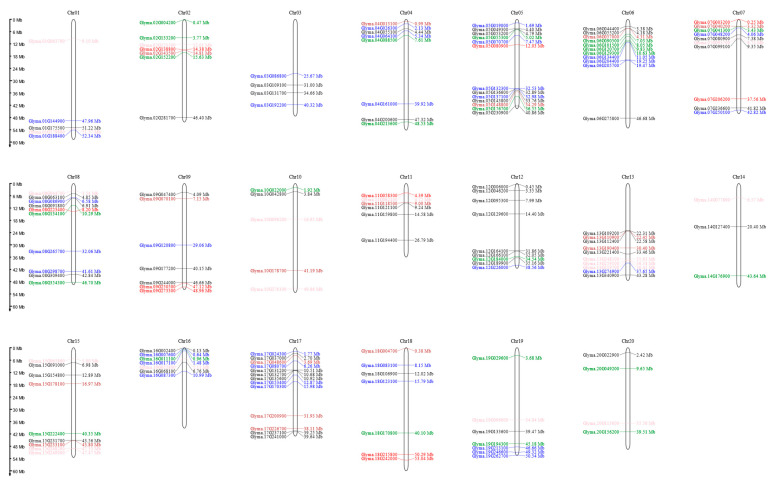
Chromosomal distribution of soybean homologs of rice grain weight-related genes. The vertical axis represents the physical length of each chromosome in megabases (Mb), and the horizontal axis indicates chromosome numbers (Chr01–Chr20). Gene loci are color-coded based on the predicted functional pathways of their corresponding rice homologs: black, hormone signaling and homeostasis; pink, ubiquitin-proteasome pathway; brown, G protein signaling; red, MAPK signaling pathway; blue, transcriptional regulation; and green, other size-related functions.

Given the relatively comprehensive understanding of the genetic regulatory mechanisms governing rice grain size [4], we selected 78 functionally validated rice grain weight-related genes as query sequences. Through BLAST analysis of their amino acid sequences, we retained the two soybean homologs exhibiting the highest sequence similarity to each query. After removing sequences with missing data or duplicates, a total of 155 soybean homologs were obtained. These genes are distributed across 20 chromosomes, with the highest densities on chromosomes 5 and 17, each harboring 13 genes, and the lowest on chromosome 14 with only 3 genes. The remaining chromosomes contain between 4 and 11 genes. Based on their predicted biological processes and molecular functions, the identified homologs were classified into six categories: hormone signaling and homeostasis (53 genes), the ubiquitin–proteasome pathway (18 genes), G protein signaling (16 genes), the MAPK signaling pathway (10 genes), transcriptional regulation (34 genes), and other seed size-related functions (24 genes) (Figure 1).

### 3.2. Analysis and Screening of Soybean Homologous Genes Associated with Seed Weight

Based on published soybean resequencing data [17], haplotype analysis of the identified homologous genes revealed 40 candidate genes that exhibited significant differences in seed weight among haplotypes, corresponding to 34 rice grain weight-related genes. Among these, 32 genes contained one to three variant sites, while the remaining eight harbored four to seven variant sites. Frequency analysis of the superior haplotype Hap1 revealed that its frequency in 22 genes progressively increased from wild accessions to landraces to cultivated varieties, indicating positive selection during breeding. Integration with transcriptome data [18,19] revealed that the seed expression levels of 21 genes were significantly correlated with seed weight, with 19 positively correlated and two negatively correlated (Table 1). Through comprehensive screening, we identified 12 key genes that simultaneously met three criteria: significant differences in seed weight between haplotypes, fixation of high-seed-weight haplotypes in cultivated varieties, and significant correlations between seed expression levels and seed weight. These genes represent priority targets for subsequent functional studies.

### 3.3. Expression Profiling and Candidate Gene Screening in Soybean

**Figure 2:**
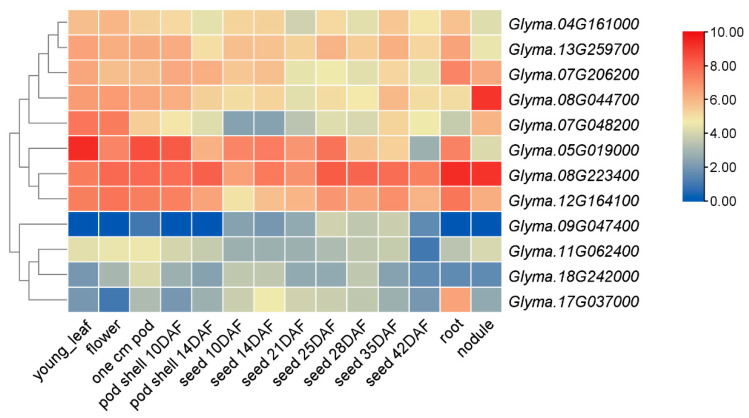
Expression profile analysis of soybean homologous genes.

Expression profiles were generated and clustered for the 12 candidate genes, revealing two major clusters: one comprising eight genes with high expression across multiple tissues, and another consisting of four genes with overall lower expression levels (Figure 2). The highly expressed cluster included homologs of *CLG1* (*Glyma.08G044700*), *GW7* (*Glyma.04G161000*), *OsARF4* (*Glyma.12G164100*), *OsMAPK6* (*Glyma.07G206200*), *OsMKK4* (*Glyma.08G223400*), *OsSPL18* (*Glyma.05G019000*), *RAV6* (*Glyma.07G048200*), and *OsUBP15* (*Glyma.13G259700*). Among these, *UBP15*, a key member of the ubiquitin-specific protease family, has been well documented to regulate seed size in Arabidopsis, rice, and Brassica napus. However, no studies on UBP15 in soybean have been reported to date. Therefore, this study focused on the soybean homologs of OsUBP15 for subsequent analysis.

### 3.4. Identification of UBP15 Homologs in Soybean

**Figure 3:**
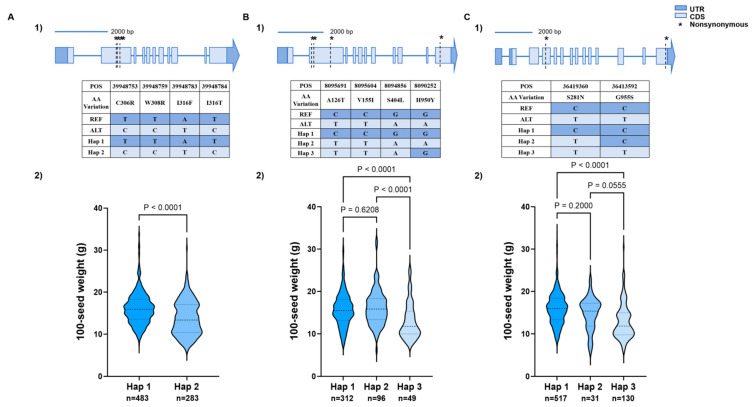
Haplotype analysis and phenotypic variation of *GmUBP5*, *GmUBP11*, and *GmUBP33*. (**A**) Gene structure, variant sites, and haplotype analysis of *GmUBP5*. (**B**) Gene structure, variant sites, and haplotype analysis of *GmUBP11*. (**C**) Gene structure, variant sites, and haplotype analysis of *GmUBP33*. In each panel, 1) shows gene structure, variant sites, and haplotype analysis; 2) shows phenotypic distribution of 100-seed weight among different haplotype groups. Different colors represent different haplotypes. Statistical significance was determined by one-way ANOVA with post-hoc test and two-tailed Student’s *t*-test. *p* < 0.05 was considered statistically significant. n indicates the number of accessions analyzed.

Ubiquitination plays a crucial role in plant responses to abiotic stress and the regulation of growth and development [20]. To investigate the association between *OsUBP15* homologs and seed weight in soybean, we identified 11 homologs in the soybean genome using the rice OsUBP15 protein sequence as a query. Variant site analysis revealed that four genes, *GmUBP5*, *GmUBP11*, *GmUBP33*, and *GmUBP40*, contained two to five non-synonymous mutation sites (Table 2). Further haplotype analysis indicated significant differences in seed weight among haplotypes for *GmUBP5*, *GmUBP11*, and *GmUBP33*, suggesting that genetic variation in these genes may contribute to soybean seed weight regulation (Figure 3). Therefore, these three genes were selected for further investigation in this study.

### 3.5. Haplotype Frequency Analysis of GmUBP5, GmUBP11, and GmUBP33

**Figure 4:**
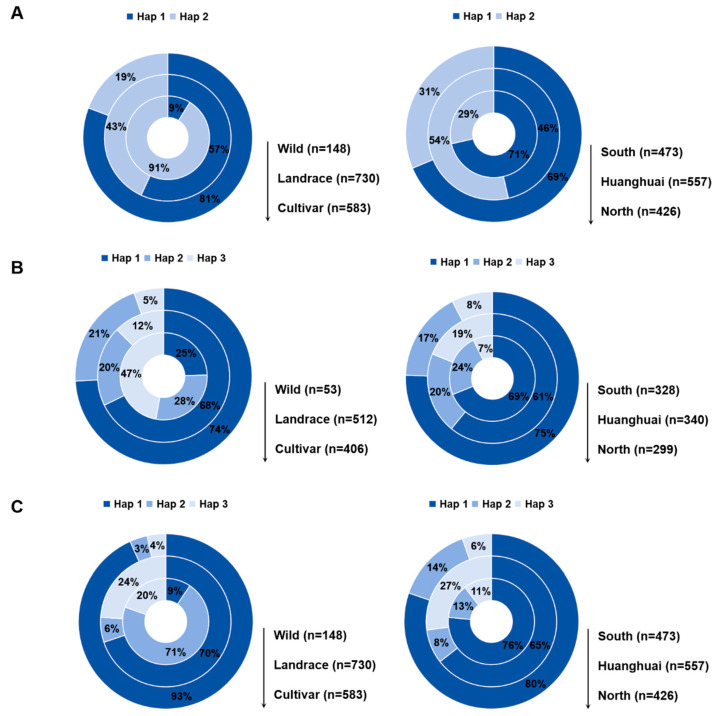
Haplotype frequency and geographic distribution of *GmUBP5*, *GmUBP11*, and *GmUBP33* in the soybean natural population. (**A**) Haplotype frequency and geographic distribution of *GmUBP5*. (**B**) Haplotype frequency and geographic distribution of *GmUBP11*. (**C**) Haplotype frequency and geographic distribution of *GmUBP33*.

To assess the breeding value of *GmUBP5*, *GmUBP11*, and *GmUBP33*, we analyzed the frequency distribution of their haplotypes (Figure 4). The elite haplotype (Hap1) exhibited a marked gradient increase across soybean populations. In wild accessions, Hap1 accounted for only 9% to 25% of individuals, whereas its frequency increased substantially to 57% to 70% in landraces, and further rose to 74% to 93% in improved cultivars. These results indicate that the elite Hap1 haplotype has been progressively enriched during domestication and continues to be selected in modern breeding programs.

We next examined the geographic distribution of elite haplotypes for each gene across major soybean-producing regions in China (Figure 4). For *GmUBP5*, the elite haplotype maintained high frequencies in varieties from both the southern and northern regions (69% to 71%), but showed a relatively lower frequency (46%) in the Huang-Huai region. For GmUBP11 and GmUBP33, the elite haplotypes exhibited consistently high frequencies across all three regions, ranging from 61% to 80%. These findings suggest that the elite haplotypes of *GmUBP11* and *GmUBP33* have been broadly utilized across diverse ecological zones, while the elite haplotype of *GmUBP5* may exhibit regional preference.

### 3.6. Joint Haplotype Analysis of GmUBP5, GmUBP11, and GmUBP33

**Figure 5:**
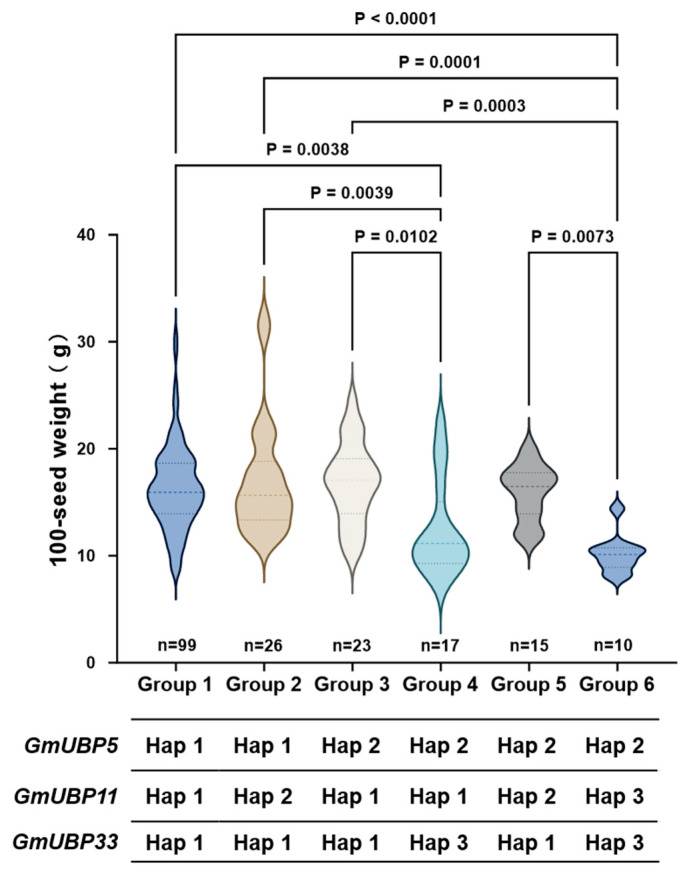
Joint haplotype analysis of *GmUBP5*, *GmUBP11*, and *GmUBP33* and their association with seed weight. Six distinct haplotype combinations (Groups 1–6) were identified. Different colors represent different haplotype combination groups. Statistical significance was determined by one-way ANOVA with post-hoc test. *p* < 0.05 was considered statistically significant.

To elucidate the synergistic regulatory effects of *GmUBP5*, *GmUBP11*, and *GmUBP33* on soybean seed weight, we performed a joint haplotype analysis of these three genes (Figure 5). Excluding the extremely rare natural haplotypes, these three factors jointly account for six distinct haplotype combinations (Groups 1–6). Groups 1, 2, and 3, which consisted of two or three elite haplotypes, exhibited the highest average seed weight. In contrast, Groups 4 and 6, comprising one or two low-seed-weight haplotypes, showed the lowest average values. Group 1 represented the optimal combination, carrying the elite haplotype of each gene (*GmUBP5^Hap1^-GmUBP11^Hap1^-GmUBP33^Hap1^*), and displayed the highest frequency among the tested accessions.

Significant differences in seed weight were observed between Groups 1, 2, and 3 compared to Groups 4 and 6, indicating a synergistic effect of these three genes in regulating seed weight. These results demonstrate that combining elite and low-seed-weight alleles from different genes can cooperatively influence soybean seed weight, providing a theoretical basis for polygenic aggregation breeding in soybean (Table 3).

### 3.7. Experimental Validation of Subcellular Localization for GmUBP5, GmUBP11, and GmUBP33

**Figure 6:**
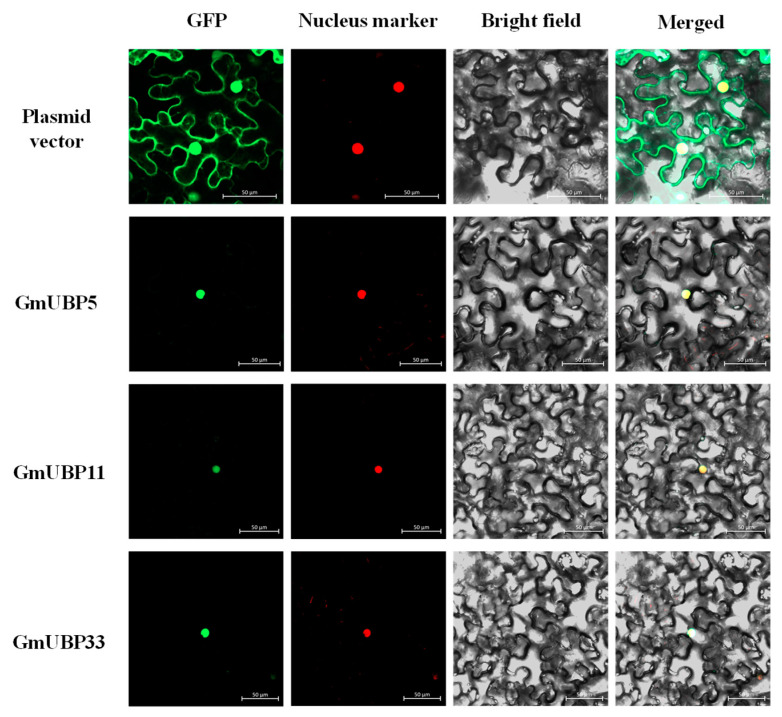
Subcellular localization of GmUBP5, GmUBP11, and GmUBP33 in tobacco leaf epidermal cells. GFP fluorescence (green dots) indicates the localization of the target proteins, the red dots repre-sent the nucleus marker, and the yellow dots in the merged images show co-localization of the target protein with the nucleus. Scale bar = 50 μm.

To determine the subcellular localization of GmUBP5, GmUBP11, and GmUBP33, we performed transient expression assays in tobacco (*Nicotiana benthamiana*) leaf epidermal cells using the 35S-driven pCEP01-linker-GFP vector as a control (Figure 6). Co-localization with a red fluorescent nuclear marker confirmed the nuclear localization of GmUBP5, GmUBP11, and GmUBP33. Confocal microscopy revealed that the GmUBP5-GFP, GmUBP11-GFP, and GmUBP33-GFP fusion proteins were exclusively localized to the nucleus. These results suggest that the three GmUBP proteins may act as deubiquitinating enzymes within the nucleus, where they regulate the expression of genes involved in soybean seed development.

### 3.8. Validation of Associations Between Markers and 100-Seed Weight Phenotype

Based on six non-synonymous SNP sites in *GmUBP5*, *GmUBP11*, and *GmUBP33* that were significantly associated with seed weight in soybean, we developed corresponding kompetitive allele-specific PCR (KASP) markers. These markers were used to genotype 54 soybean accessions. Among the tested accessions, 16 carried all six high-seed-weight alleles, with hundred-seed weights ranging from 17.72 to 33.73 g (mean = 24.57 g). Seventeen accessions carried four high-seed-weight alleles, exhibiting hundred-seed weights from 12.64 to 24.39 g (mean = 18.47 g). Eleven accessions carried three high-seed-weight alleles, with hundred-seed weights ranging from 9.19 to 21.54 g (mean = 15.33 g). Ten accessions carried no high-seed-weight alleles, with hundred-seed weights ranging from 7.78 to 22.17 g (mean = 13.67 g) (Table 4).

**Figure 7:**
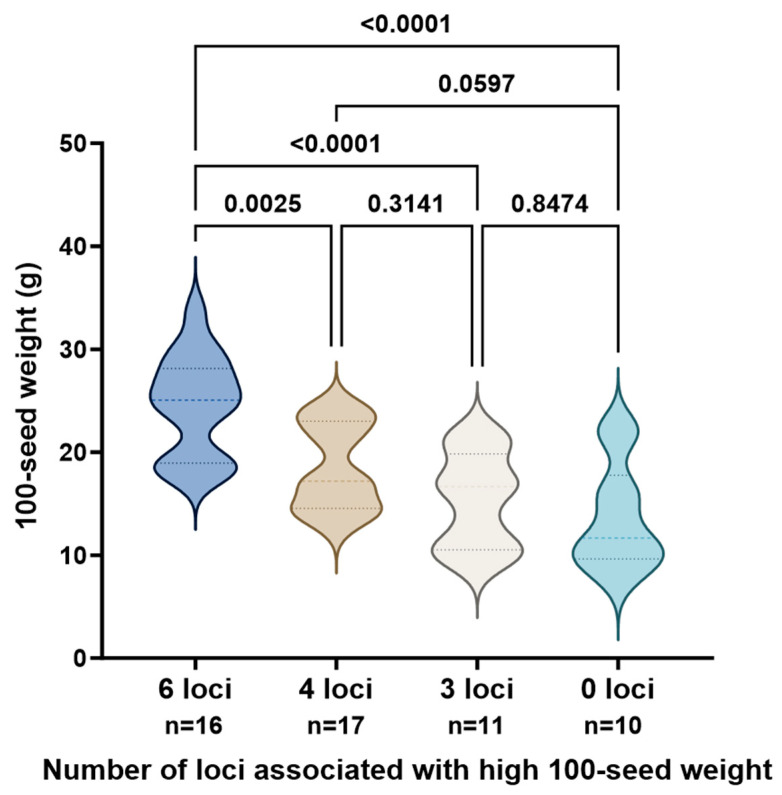
KASP marker-based genotyping and phenotypic validation in soybean accessions. Different colors represent groups with different numbers of associated loci. Statistical significance was determined by one-way ANOVA with post-hoc test. *p* < 0.05 was considered statistically significant.

Accessions carrying all six advantageous alleles exhibited significantly higher seed weights than those carrying four, three, or none of the alleles. These results demonstrate that the KASP marker-based system efficiently distinguishes soybean germplasm with contrasting hundred-seed weight phenotypes (Figure 7).

## 4. Discussion

### 4.1. Identification of Genes Associated with Soybean Seed Weight

Orthologous genes generally retain functional conservation across species [21,22], enabling efficient identification of candidate genes for target traits based on known functional genes. For instance, four homologs of Arabidopsis *WOX1* have been identified in soybean, and mutations in these genes lead to narrower leaves [23]. Similarly, knocking out homologs of key genes involved in Arabidopsis triacylglycerol synthesis has resulted in novel soybean germplasm enriched in diacylglycerol and lecithin [24]. However, this strategy has been relatively underexplored in studies on soybean seed weight.

Given that the molecular network regulating grain size in rice has been systematically characterized [25], the present study used 78 functionally validated rice grain weight regulators as queries to perform BLAST searches against the soybean genome, aiming to identify candidate genes associated with seed weight. Through sequence alignment and haplotype analysis, 40 candidate genes showing significant associations with seed weight were identified, most of which have not been previously reported, thus expanding the genetic resource pool for seed weight regulation in soybean. Among these, 22 genes exhibited large-seed haplotypes that were preferentially selected during breeding, and 21 genes showed a significant correlation between transcript abundance and seed weight. Further filtering based on expression profiles narrowed the candidates to eight genes that were selected during breeding, displayed significant expression-seed weight correlations, and were highly expressed in seeds. These eight genes all positively regulate soybean seed weight and are involved in diverse biological processes, including grain filling and morphogenesis, seed development, auxin signaling, MAPK cascades, transcriptional regulation, and ubiquitination pathways, thereby providing promising targets for genetic improvement of seed weight in soybean.

### 4.2. Functional Conservation of UBP15 and Its Selection and Adaptation in Soybean

The ubiquitin-proteasome pathway is a key regulatory mechanism underlying crop seed development. Several soybean seed weight-related genes, including GmSW17 and GmSMS6, have been implicated in ubiquitination processes [26,27]. Ubiquitin-specific proteases (UBPs) constitute the largest subfamily of deubiquitinating enzymes (DUBs) and have been reported to regulate seed size in various plant species. Among them, UBP15 has been functionally characterized in *Arabidopsis*, rice, and rapeseed (*Brassica napus*), yet its role in soybean remains largely unknown.

In this study, we used the rice UBP15 amino acid sequence as a query to identify homologous genes in the soybean genome, resulting in the identification of *GmUBP5*, *GmUBP11*, and *GmUBP33*. Haplotype analysis revealed that all three genes carry non-synonymous mutations that are significantly associated with seed weight, suggesting these sites may play critical roles in regulating soybean seed weight. The large-seed haplotypes of these genes were found at high frequencies in natural populations and appear to have been preferentially selected during breeding.

Geographic distribution analysis showed that the high-seed-weight haplotypes of *GmUBP11* and *GmUBP33* are predominant across all three major soybean production regions in China—Southern China, the Huang-Huai region, and Northern China—indicating broad ecological adaptability and potential utility in wide-area high-yield breeding. In contrast, the high-seed-weight haplotype of *GmUBP5* exhibited a frequency of only 46% in the Huang-Huai region, significantly lower than that in the Southern and Northern regions. This suggests that this haplotype remains underexploited in current soybean germplasm from the Huang-Huai region, highlighting its considerable potential for future breeding applications.

### 4.3. Cumulative Effects of GmUBP5, GmUBP11, and GmUBP33 on Seed Weight Regulation

Stacking beneficial mutations is an effective strategy for enhancing crop traits. In soybean, edited lines derived from the *GmAHAS* gene family—which encodes acetohydroxyacid synthase—exhibit herbicide resistance levels positively correlated with the number of mutated genes, with lines carrying mutations in all three genes showing additive effects and broad-spectrum resistance [28]. Similarly, the soybean oil and protein content regulator *GmSop20* and its downstream target *GmSWEET10a* act additively; their combined effect on improving the seed oil-to-protein ratio is greater than that of either gene alone [29]. These findings suggest that joint haplotype analysis based on multiple favorable loci is an effective approach for dissecting the synergistic and additive effects of gene combinations on target traits.

In this study, we observed significant additive effects among GmUBP5, GmUBP11, and GmUBP13 in regulating soybean seed weight. Pyramiding multiple high-seed-weight haplotypes resulted in a substantial increase in seed weight. Among all combinations, *GmUBP5^Hap1^-GmUBP11^Hap1^-GmUBP33^Hap1^* occurred at the highest frequency (52.11%) in the germplasm panel, suggesting that this combination may have already been selected in soybean breeding and represents a promising target for polygenic pyramiding. Elucidating the underlying synergistic mechanisms may open new avenues for molecular design breeding targeting seed size and yield.

Furthermore, we developed a KASP marker system based on six core non-synonymous polymorphisms in *GmUBP5*, *GmUBP11*, and *GmUBP33*. This system efficiently identifies high-seed-weight alleles, and the number of favorable alleles carried is positively correlated with seed weight. With its low cost and high throughput, this marker system is well suited for early-stage selection in breeding populations and can accelerate the development of large-seeded soybean varieties.

### 4.4. Potential Functional Redundancy of GmUBP5, GmUBP11, and GmUBP33

Protein subcellular localization critically influences protein function, stability, and abundance [30]. Ubiquitination occurs at various subcellular compartments, and its specific site of action is often closely associated with the underlying biological process. For instance, the wheat E3 ubiquitin ligase TaE3V1 interacts with its partner TaVRN1 at the plasma membrane to regulate vernalization via ubiquitination [31], whereas Arabidopsis HOS1 mediates the ubiquitination and degradation of the transcription factor SPL9 in the nucleus, thereby modulating flowering time under salt stress [32].

In this study, GmUBP5, GmUBP11, and GmUBP33 were all localized to the nucleus, suggesting that they may regulate the transcriptional activity of seed development-related genes through deubiquitination, thereby affecting soybean seed weight. Soybean has undergone two whole-genome duplication events during its evolution, resulting in numerous multi-copy genes [33]. In gene families shaped by polyploidization or duplication, functional redundancy is common among homologous members [34]. Consistent with this, GmUBP5, GmUBP11, and GmUBP33 all localize to the nucleus and are significantly associated with seed weight, raising the possibility of functional redundancy among them. Future studies examining their expression patterns across seed developmental stages and generating single, double, and triple mutants via gene editing will help clarify their functional relationships through comparative seed weight analyses.

## 5. Conclusions

In this study, we identified 155 soybean homologs of 78 functionally validated rice grain weight-related genes. By integrating resequencing data, seed weight phenotypes from 1100 soybean accessions, and transcriptomic profiles, we performed multidimensional screening, including haplotype analysis, haplotype frequency analysis, and expression profiling—and prioritized eight candidate genes that exhibited high expression in seeds, significant correlation with seed weight, and evidence of selection during domestication. Focusing on the ubiquitin-related homolog *OsUBP15*, we identified three genes: *GmUBP5*, *GmUBP11*, and *GmUBP33*, that showed significant differences in seed weight between haplotypes. The elite haplotypes of these genes were confirmed to have been fixed during breeding and to possess regional adaptability. Joint haplotype analysis revealed significant additive effects among the three genes, and subcellular localization assays demonstrated that their protein products localize to the nucleus. Based on key non-synonymous SNP sites, we developed KASP molecular markers that efficiently distinguish large-seeded from small-seeded germplasm. Together, these findings provide valuable genetic resources and practical molecular tools for the genetic improvement of seed weight in soybean, and offer theoretical foundations and technical support for high-yield molecular breeding.

## Figures and Tables

**Table 1:** Analysis results of homologous genes with significant haplotype difference. Spearman’s correlation analysis was used to evaluate the relationship between gene expression level and 100-seed weight. Correlation coefficients (r) and corresponding *p*-values are shown. *p* < 0.05 was considered statistically significant. Identity and E-value were derived from BLASTp alignment against rice proteins.

Homologous Gene ID	Rice Gene	Identity/%	E-Value	Variation Type and Counts	Number of Haplotype	Large-Seed Haplotype Selected	Expression-100-Seed Weight Correlation
*Glyma.07G093700*	*OsCLG1*	64	0	Nonsynonymous (3)	2	Neutral	No correlation (*r* = 0.147, *p* = 0.135)
*Glyma.08G044700*	*OsCLG1*	73	0	Nonsynonymous (3)	3	Selected	Positive correlation (*r* = 0.185, *p* = 0.005)
*Glyma.16G068100*	*OsD11*	55	9.44 × 10^−178^	Nonsynonymous (2)	3	Selected	No correlation (*r* = 0.061, *p* = 0.337)
*Glyma.02G004200*	*OsDEP2*	47	2.39 × 10^−21^	Nonsynonymous (7)	4	Neutral	No correlation (*r* = −0.009, *p* = 0.924)
*Glyma.09G273300*	*OsFLR1*	67	0	Nonsynonymous (1)	2	Neutral	Positive correlation (*r* = 0.167, *p* = 0.004)
*Glyma.06G101500*	*OsGF14f*	84	8.49 × 10^−159^	Nonsynonymous (1)	2	Neutral	Positive correlation (*r* = 0.331, *p* < 0.001)
*Glyma.17G132700*	*OsGL10*	58	1.72 × 10^−61^	Nonsynonymous (3)	2	Selected	No correlation (*r* = −0.075, *p* = 0.213)
*Glyma.07G236600*	*OsGS5*	64	0	Nonsynonymous (3)	2	Neutral	Positive correlation (*r* = 0.251, *p* = 0.002)
*Glyma.17G037000*	*OsGS5*	64	0	Nonsynonymous (1)	2	Selected	Positive correlation (*r* = 0.163, *p* = 0.021)
*Glyma.05G049300*	*OsGS6*	55	3.69 × 10^−124^	Nonsynonymous (6)	3	Selected	No correlation (*r* = 0.028, *p* = 0.700)
*Glyma.05G055300*	*OsGSA1*	47	1.63 × 10^−81^	frameshift (1)	2	Selected	No correlation (*r* = −0.033, *p* = 0.569)
*Glyma.19G029600*	*OsGSA1*	53	2.14 × 10^−47^	Nonsynonymous (1), stopgain (1)	3	Selected	No correlation (*r* = 0.042, *p* = 0.488)
*Glyma.12G129600*	*OsGSK2*	90	0	Nonsynonymous (1)	2	Neutral	Positive correlation (*r* = 0.156, *p* = 0.019)
*Glyma.03G131700*	*OsGW6*	78	4.99 × 10^−33^	Nonsynonymous (1)	2	Neutral	No correlation (*r* = −0.019, *p* = 0.810)
*Glyma.04G161000*	*OsGW7*	62	3.10 × 10^−39^	Nonsynonymous (3)	3	Selected	Positive correlation (*r* = 0.183, *p* = 0.001)
*Glyma.06G204400*	*OsGW7*	62	2.06 × 10^−36^	Nonsynonymous (4)	4	Selected	No correlation (*r* = −0.224, *p* = 0.103)
*Glyma.06G205700*	*OsGW8*	70	3.6 × 10^−31^	Nonsynonymous (2), frameshift (1)	2	Neutral	No correlation (*r* = 0.098, *p* = 0.233)
*Glyma.11G062400*	*OsHDR3*	66	2.32 × 10^−111^	Nonsynonymous (3)	3	Selected	Positive correlation (*r* = 0.130, *p* = 0.031)
*Glyma.01G063700*	*OsIPA1*	75	5.49 × 10^−33^	Nonsynonymous (4)	3	Neutral	No correlation (*r* = −0.101, *p* = 0.135)
*Glyma.12G164100*	*OsARF4*	68	5.35 × 10^−169^	Nonsynonymous (1)	2	Selected	Positive correlation (*r* = 0.129, *p* = 0.026)
*Glyma.02G281700*	*OsARF6*	84	0	Nonsynonymous (1)	2	Neutral	Positive correlation (*r* = 0.169, *p* = 0.002)
*Glyma.17G153400*	*OsbZIP76*	86	0	Nonsynonymous (2)	3	Neutral	No correlation (*r* = 0.092, *p* = 0.130)
*Glyma.19G194300*	*OsCEN2*	79	2.6 × 10^−101^	Nonsynonymous (4)	4	Neutral	No correlation (*r* = 0.123, *p* = 0.103)
*Glyma.10G098200*	*OsDA1*	82	4.85 × 10^−38^	Nonsynonymous (1), frameshift (1)	3	Neutral	No expression in seeds
*Glyma.19G246600*	*OsGIF1*	74	3.54 × 10^−20^	Nonsynonymous (2)	2	Neutral	No correlation (*r* = 0.085, *p* = 0.174)
*Glyma.20G156200*	*OsIQD14*	83	7.91 × 10^−12^	Nonsynonymous (1)	2	Selected	No correlation (*r* = 0.098, *p* = 0.324)
*Glyma.07G206200*	*OsMAPK6*	93	0	Nonsynonymous (2), frameshift (1)	8	Selected	Positive correlation (*r* = 0.275, *p* < 0.001)
*Glyma.08G223400*	*OsMKK4*	63	3.85 × 10^−128^	Nonsynonymous (1)	2	Selected	Positive correlation (*r* = 0.215, *p* < 0.001)
*Glyma.10G042800*	*OsPIL15*	80	3.38 × 10^−29^	Nonsynonymous (6)	4	Neutral	Negative correlation (*r* = −0.230, *p* = 0.007)
*Glyma.09G047400*	*OsPUP4*	51	9.37 × 10^−62^	Nonsynonymous (2)	2	Selected	Positive correlation (*r* = 0.323, *p* < 0.001)
*Glyma.17G170300*	*OsSNB*	81	1.59 × 10^−84^	Nonsynonymous (2)	2	Neutral	Positive correlation (*r* = 0.123, *p* = 0.038)
*Glyma.05G019000*	*OsSPL18*	71	1.37 × 10^−31^	Nonsynonymous (3)	3	Selected	Positive correlation (*r* = 0.138, *p* = 0.037)
*Glyma.09G250500*	*OsWRKY53*	78	1.43 × 10^−22^	Nonsynonymous (7)	3	Selected	No correlation (*r* = −0.047, *p* = 0.583)
*Glyma.18G242000*	*OsWRKY53*	78	1.81 × 10^−22^	Nonsynonymous (5)	5	Selected	Positive correlation (*r* = 0.201, *p* = 0.003)
*Glyma.09G120800*	*OsPOW1*	27	8.42 × 10^−03^	Nonsynonymous (2)	3	Selected	No expression in seeds
*Glyma.07G048200*	*OsRAV6*	87	1.88 × 10^−61^	Nonsynonymous (1)	2	Selected	Positive correlation (*r* = 0.121, *p* = 0.029)
*Glyma.16G017100*	*OsRAV6*	87	3.22 × 10^−62^	Nonsynonymous (2)	2	Neutral	Positive correlation (*r* = 0.215, *p* < 0.001)
*Glyma.02G133200*	*OsTGW2*	66	3.11 × 10^−60^	Nonsynonymous (3)	4	Selected	No correlation (*r* = 0.060, *p* = 0.427)
*Glyma.13G259700*	*OsUBP15*	60	0	Nonsynonymous (2)	3	Selected	Positive correlation (*r* = 0.217, *p* < 0.001)
*Glyma.19G096600*	*OsWG1*	73	2.26 × 10^−33^	Nonsynonymous (1)	2	Neutral	Negative correlation (*r* = −0.214, *p* = 0.001)

**Table 2:** List of *UBP15* homologous genes. Data include gene ID, number of nonsynonymous mutation sites, BLAST E-value, and protein sequence identity compared with *OsUBP15*.

Gene Name	Gene ID	Number of Nonsynonymous Sites	E-Value	Identity/%
*GmUBP3*	*Glyma.02G040900*	0	4.05 × 10^−13^	50
*GmUBP5*	*Glyma.02G213400*	4	1.52 × 10^−11^	70
*GmUBP9*	*Glyma.04G058300*	0	2.65 × 10^−7^	61
*GmUBP11*	*Glyma.04G091700*	4	1.04 × 10^−116^	45
*GmUBP14*	*Glyma.06G059000*	0	4.19 × 10^−7^	53
*GmUBP16*	*Glyma.06G093500*	0	1.84 × 10^−109^	51
*GmUBP33*	*Glyma.13G259700*	2	0	60
*GmUBP36*	*Glyma.14G105700*	0	2.93 × 10^−6^	58
*GmUBP39*	*Glyma.14G181100*	0	6.22 × 10^−118^	51
*GmUBP40*	*Glyma.15G248200*	5	0	59
*GmUBP46*	*Glyma.17G220700*	0	2.84 × 10^−6^	58

**Table 3:** Phenotypic variation of 100-seed weight among different joint haplotype groups.

Joint Haplotype Group	Min (g)	Max (g)	Mean ± SD (g)
Group 1	8.52	30.51	16.34 ± 3.94
Group 2	11.48	32.13	17.06 ± 5.31
Group 3	10.20	24.35	16.82 ± 3.75
Group 4	7.28	22.46	12.43 ± 4.45
Group 5	11.60	20.07	15.92 ± 2.62
Group 6	8.01	14.4	10.15 ± 1.80

**Table 4:** Statistical comparison of 100-seed weight among combinations with different numbers of associated loci.

Number of Favorable Alleles	Min (g)	Max (g)	Mean ± SD (g)
6 loci	17.72	33.73	24.57 ± 4.92
4 loci	12.64	24.39	18.47 ± 4.17
3 loci	9.19	21.54	15.33 ± 4.59
0 loci	7.78	22.17	13.67 ± 5.14

## Data Availability

The data that support the findings of this study are available from the corresponding authors upon reasonable request.

## Supplementary Materials

The following supporting information can be downloaded at: https://www.mdpi.com/article/10.3390/biology15090727/s1, Table S1: Gene IDs and Functional Annotations; Table S2: List of Primers; Table S3: KASP Analysis Results.
